# The Effects of Inactivation of Pedunculopontine Tegmental Nucleus by Cobalt (II) Chloride on Cardiovascular Responses in Hemorrhagic Hypotensive Rats

**DOI:** 10.32598/bcn.9.10.315

**Published:** 2019-05-01

**Authors:** Reza Mohebbati, Mahmoud Hosseini, Majid Khazaei, Abolfazl Khajavirad, Mohammad Naser Shafei

**Affiliations:** 1. Department of Physiology, School of Medicine, Mashhad University of Medical Sciences, Mashhad, Iran.; 2. Department of Neurocognitive Sciences, Psychiatry and Behavioral Sciences Research Center, Mashhad University of Medical Sciences, Mashhad, Iran.; 3. Neurogenic Inflammation Research Center, Mashhad University of Medical Sciences, Mashhad, Iran.

**Keywords:** Hemorrhage, Cobalt (II) chloride, Blood pressure, Heart rate, Pedunculopontine Tegmental nucleus (PPT)

## Abstract

**Introduction:**

Based on the evidence, the Pedunculopontine Tegmental nucleus (PPT) is involved in cardiovascular function regulation. In this study, the probable role of PPT on cardiovascular parameters in the hypotension induced by Hemorrhage (HEM) was evaluated.

**Methods:**

The study rats were divided up into 5 groups: 1. Control (Saline); 2. Cobalt(II) chloride (CoCl_2_); 3. HEM; 4. Saline+HEM; and 5. CoCl_2_+HEM. Their right and left femoral arteries were cannulated for recording the cardiovascular responses and blood withdrawal, respectively. Saline and CoCl_2_ were microinjected into the PPT using the stereotaxic apparatus. Maximum changes of Systolic Blood Pressure (SBP), Mean Arterial Pressure (MAP), and the Heart Rate (HR) after the microinjection of CoCl_2_ in normal and Hemorrhage conditions were recorded. Changes of SBP, MAP, and HR were calculated over time at 5-min intervals and compared with those of the control and HEM groups using repeated measures ANOVA. The Independent sample t-test was used to compare the changes in cardiovascular parameters between the control and HEM groups at 0 and 20 min after Hemorrhage.

**Results:**

The changes in SBP, MAP, and HR in the CoCl_2_ group were not significantly different from those in the control group. In the HEM group, the SBP and MAP changes significantly decreased (P<0.001) and HR changes significantly increased (P<0.001) compared to those parameters in the control group. In the CoCl_2_+HEM group, SBP and MAP changes were significantly attenuated compared to those in the HEM group (P<0.05) and HR changes induced by Hemorrhage decreased compared to that in the HEM group (P<0.01).

**Conclusion:**

Our results indicate that the PPT has no effects on normal cardiovascular parameters. However, it could modulate cardiovascular responses induced by Hemorrhage.

## Highlights

- Hemorrhage induces hypotension and tachycardia.
- Inactivation of the Pedunculopontine Tegmental nucleus (PPT) in the normotensive rats does not significantly affect cardiovascular responses.
- Inactivation of the PPT during hemorrhage can significantly attenuate hypotension and tachycardia induced by hemorrhage.

## Plain Language Summary

Hemorrhage or bleeding is a serious condition characterized by a reduction in blood volume and diminishes blood supply to the tissue. If bleeding continues, it can damage organs and even cause death. Hypovolemic hemorrhage evokes several brain regions to send signals and maintain cardiovascular parameters at or near normal levels. One of these areas is the Pedunculopontine Tegmental nucleus (PPT) that is involved in a variety of functions such as locomotion, sleep, and regulation of respiratory and cardiovascular functions. Because the relationship of the PPT with brain areas involved in cardiovascular regulation in hemorrhage has been already proven, we presume that this nucleus precipitates in cardiovascular regulation during hemorrhage. Therefore, in this study we inactivate the nucleus by injection of cobalt (II) chloride, a nonselective synapse blocker, to evaluate the possible cardiovascular effect of this nucleus in hypovolemic hemorrhage. The study animals were anesthetized, and their left femoral arteries were cannulated for the recording of systolic blood pressure, mean arterial pressure and heart rate. The right femoral artery was also cannulated for the blood withdrawal. Hemorrhage was done by removal of blood from the arterial catheter in a constant and controlled rate. Our results indicate that inactivation of PPT in normotensive rats did not significantly change cardiovascular parameters. However, inactivation of the nucleus in hypovolemic hemorrhage condition significantly attenuated hypotension and tachycardia. These results indicate that PPT could modulate cardiovascular responses caused by hemorrhage.

## 1. Introduction

Hemorrhage is a life-threatening incident that through the loss of intravascular volume can cause hypotension. Hemorrhage has two compensatory and non-compensatory phases (Evans, Ventura, Dampney, & Ludbrook, 2001). In the compensatory phase, baroreceptor reflex activates and increases the sympathetic drive to maintain the blood pressure at near normal level. If the blood loss continues, the non-compensatory phase starts, in which the sympathetic drive abruptly decreases and arterial pressure falls.

The central mechanism responsible for these phases is not completely obvious. However, the involvement of several brain regions such as Rostral Ventrolateral Medulla (RVLM), Nucleus Tractus Solitarius (NTS), Periaqueductal Grey (PAG), Paraventricular (PVN) and Caudal Ventrolateral Medulla (CVLM) have been mentioned in the regulation of cardiovascular system during Hemorrhage by immunohistochemical studies (Evans et al., 2001). In addition, the role of several brain nuclei has been determined in regulating the cardiovascular system, but their importance, such as Pedunculopontine Tegmental nucleus (PPT) in Hemorrhage is unclear.

PPT is an important region in the upper part of the brain stem that has a large number of neurons, in particular, cholinergic neurons (Benarroch, 2013; Bevan & Bolam, 1995). This nucleus is involved in a variety of functions such as locomotion (Martinez-Gonzalez, Bolam, & Mena-Segovia, 2011), awakening, REM sleep, and learning (Sleekier, Inglis, Winn, & Sahgal, 1994). PPT also contributes to the regulation of autonomic functions such as respiratory and cardiovascular functions (Pahapill & Lozano, 2000). Plowey et al. (2004) reported that inactivation of PPT with cobalt(II) chloride (CoCl_2_), a nonselective synapse blocker, significantly attenuated the respiratory reflex to exercise and proposed that PPT had a central command function for the adjustment of respiratory responses during conditions such as exercise and arousal (Plowey & Waldrop, 2004).

Topchiy, et al. (2010) also reported that microinjection of glutamate into PPT evoked several responses such as tachypnea, apnea, increase in blood pressure, and sinus tachycardia. A cholinergic projection from the PPT to RVLM, a critical area involved in the regulation of the central cardiovascular system, has been also reported in a previous study (Yasui, Cechetto, & Saper, 1990).

The inhibitory effect of the nitrergic system of PPT on the cardiovascular system has also been demonstrated in our recent study (Shafei, Nikyar, Hosseini, Niazmand, & Paseban, 2017). Although the contribution of PPT in cardiovascular regulation has been shown, its cardiovascular effect(s) during Hemorrhage has not been assessed. Because the relationship of PPT with several brain areas involved in cardiovascular regulation such as RVLM, NTS, and PAG (Steininger, Rye, & Wainer, 1992; Topchiy et al., 2010; Yasui et al., 1990) has been mentioned, we speculate that this nucleus may be involved in the regulation of cardiovascular responses during hypotension due to Hemorrhage.

To evaluate this hypothesis, PPT in rats was temporarily inactivated by CoCl_2_, a nonselective synapse blocker, then their cardiovascular responses in the normal and Hemorrhage conditions were evaluated. By blocking calcium pre-synaptic influx, CoCl_2_ inhibits the release of neurotransmitter and inactivates the function of a nucleus (Granjeiro et al., 2012).

## 2. Methods

### 2.1. Animals and procedure

The study was performed on 40 male Wistar rats. Anesthesia was induced by urethane (1.5 g/kg, IP) and a supplementary dose (0.7 g/kg). A heating lamp was used to keep the body temperature of the animal at 37°C. The left femoral artery was exposed and cannulated with a Polyethylene catheter (PE-50) filled with heparinized saline (60 u/mL). Then the catheter was connected to a blood pressure transducer and the Systolic Blood Pressure (SBP), Mean Arterial Pressure (MAP) and Heart Rate (HR) were recorded throughout the study period by a power lab system (ID instrument, Australia) (Shafei & Nasimi, 2011). The right femoral artery was also cannulated for the blood withdrawal.

For the microinjection of drugs, the animals were placed in a stereotaxic apparatus (Stoelting, USA). The scalp was chiseled and the skull was leveled between the bregma and lambda. Then a small hole was drilled in the skull. The stereotaxic coordinates of the PPT were −7.6 to −8.5 mm caudal to bregma, −1.8 to −2.2 mm lateral to the midline suture, and −6.8 to −7.8 mm ventral from the bregma according to the Paxinos and Watson (2007). CoCl_2_ (1 mM) was microinjected into the PPT nucleus by a single barreled micropipette with 35–40 μm internal diameter (Nasimi, Shafei, & Alaei, 2012).

The micropipette was connected through a PE-10 tube to a manual microinjector (Harvard) and injection was carefully performed. About 150 nL was injected in all groups in 30 seconds (Shafei et al., 2017). The study protocol was approved by the Bioethics Committee of Mashhad University of Medical Sciences (Code: IR.MUMS.REC.1394.200).

### 2.2. Hemorrhage procedure

In Hemorrhage (HEM) groups, after stabilization of the hemodynamic parameters, Hemorrhage was initiated by the withdrawal of blood from the arterial catheter (1 mL/100 g body weight) in a constant and controlled rate (0.2–0.3 mL/min for 10 min) (Ahlgren, Porter, & Hayward, 2007). After inducing Hemorrhage, the cardiovascular responses were obtained at 5-min intervals from the onset of Hemorrhage until 40 min after termination of Hemorrhage. The peak changes were also calculated 1 min and 20 min after termination of Hemorrhage (10 and 30 min after the initiation of Hemorrhage).

### 2.3. Histological procedure

To verify the microinjection site, at the end of the experiment, transcardial perfusion (100 mL of saline and then 100 mL of 10% formalin) was performed. The animal brains were removed and kept in formalin for 48 h. Then a serial slice with 60-µ thickness was prepared by microtome, and the site of injection was observed according to the Paxinos Atlas (Shafei & Nasimi, 2011).

### 2.4. Drug and animal groups

The drugs (urethane and CoCl_2_) were provided by Sigma, USA and dissolved in saline. The rats were randomly divided into 5 groups as follows: Control (saline): Microinjection of saline into the PPT; CoCl_2_: Microinjection of CoCl_2_ (1 mM) into the PPT; Hemorrhage (HEM): Induction of Hemorrhage by the withdrawal of blood over 10 min; Saline+Hemorrhage (saline+Hem): Microinjection of saline into the PPT 5 min before induction of Hemorrhage; and CoCl_2_+Hemorrhage (CoCl_2_+HEM): Microinjection of CoCl_2_ into the PPT 5 min before induction of Hemorrhage.

### 2.5. Data analysis

SBP, MAP and HR and their changes (Δ) were calculated and expressed as Mean±SEM. For the evaluation of responses in HEM groups, ΔSBP, ΔMAP, and ΔHR were calculated over time at 5-min intervals and compared the changes during the Hemorrhage (using repeated measures ANOVA). In addition, the peak changes of ΔSBP, ΔMAP, and ΔHR of all groups were also separately provided at 0 and 20 min after termination of Hemorrhage (10 and 30 min after initiation of Hemorrhage) and compared with peak changes of the control and HEM groups (using the Independent sample t-test). P<0.05 was set to indicate statistical significance.

## 3. Results

### 3.1. Cardiovascular responses of the PPT to microinjection of saline and cobalt(II) chloride

To evaluate the role of PPT at normal (baseline) cardiovascular regulation, both saline and CoCl_2_ were microinjected into the PPT nucleus in separate groups and the cardiovascular parameters were recorded for 30 min. In the saline group, the microinjection of saline did not have significant effects on Mean±SEM SBP (before: 108±2.2 mm Hg; after: 114±4.8 mm Hg), Mean±SEM MAP (before: 100.4±2.6 mm Hg; after: 98.5±3.9 mm Hg) or Mean±SEM HR (before: 330±7.9 beats/min; after: 318.5±6.4 beats/min).

**Figure 1.**
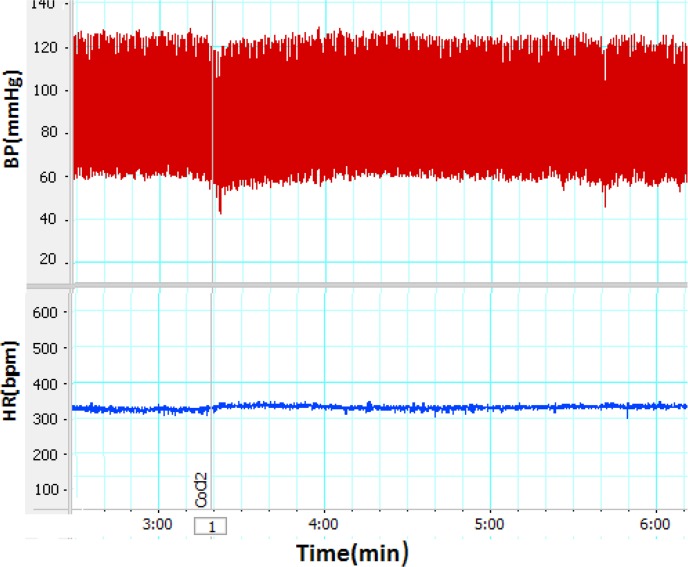
A sample of blood pressure and Heart Rate recording after inactivation of PPT nucleus with CoCl_2_ The arrow shows microinjection time.

**Figure 2.**
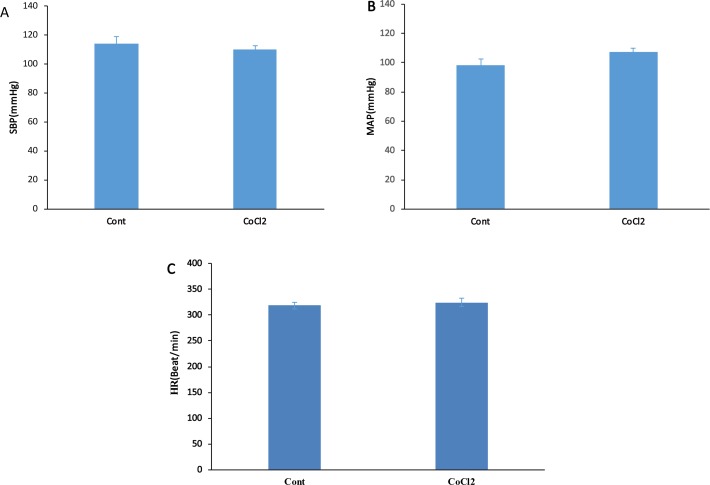
Cardiovascular responses to microinjection of CoCl_2_ into the PPT in normal rats (n=6) A. Inactivation of nucleus did not affect the peak changes of Systolic Blood Pressure (ΔSBP); B. Mean Arterial Pressure (ΔMAP); and C. Heart Rate (ΔHR) compared to the control group (using the Independent sample t-test). The data were expressed as Mean±SEM.

In CoCl_2_ group, Mean±SEM SBP (CoCl_2_: 110±2.2 mm Hg vs. saline: 114±4.8 mm Hg), Mean±SEM MAP (CoCl_2_=107.4±2.7 mm Hg vs. saline=98.5±3.9 mm Hg) or Mean±SEM HR (CoCl_2_=324.2±7.9 beats/min vs. saline: 318.5±6.4 beats/min, using the Independent sample t-test; n=8) were not significant compared to those of the saline group (Figures 1 and 2).

### 3.2. Cardiovascular responses induced by Hemorrhage in rats

**Figure 3.**
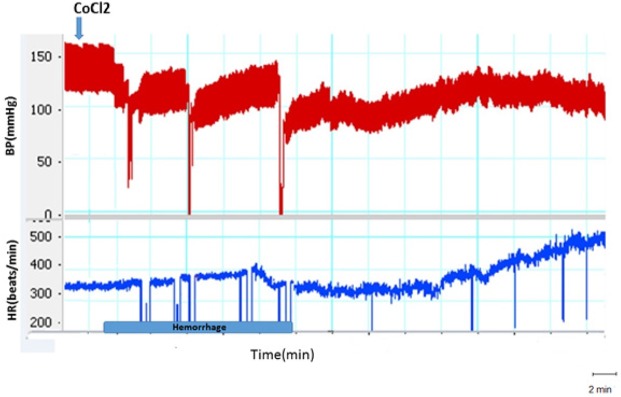
A sample of blood pressure and Heart Rate recording after inactivation of the PPT nucleus with CoCl_2_ and induction of Hemorrhage The arrow shows microinjection time.

In this group, the Hemorrhage was performed during 10 min and the cardiovascular responses were evaluated. As it is shown in Figure 3, after blood withdrawal, MAP and SBP decreased and when the blood withdrawal reached the lowest level they slowly increased and after 20 min (30 min after initiation of blood withdrawal) got stabilized but remained below the baseline levels.

**Figure 4.**
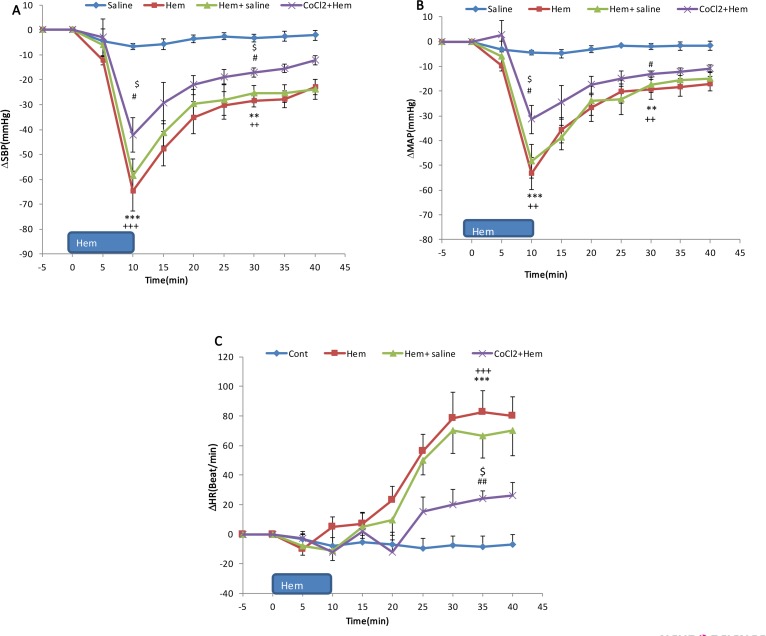
Time-course changes of Systolic Blood Pressure (ΔSBP) (A); Mean Arterial Pressure (MAP) (B); and Heart Rate (ΔHR) (C) in the experimental groups (n=6) CoCl_2_ was microinjected into the PPT 5 min before HEM. ^***^ P<0.001 peak changes of HEM group vs. control; +++ P<0.01 peak changes of HEM+ saline vs. control; # P<0.05; ## P<0.01 peak changes of HEM+CoCl_2_ vs. control; $ P<0.05 peak changes of HEM+

Time-course changes in cardiovascular parameters have been shown in Figure 4. As it is shown, ΔMAP and ΔSBP significantly decreased and ΔHR increased compared to the control group parameters over time (repeated measures ANOVA, P<0.001, n=6). Peak changes of ΔMAP, ΔSBP, and ΔHR at the end of the blood withdrawal are displayed in Figure 4.

As shown, both Mean±SEM ΔMAP and ΔSBP significantly decreased compared to those in the control group (Mean±SEM ΔSBP; HEM: −64.7±7.9 mm Hg vs. saline; −4.6±2.3 mm Hg) and (Mean±SEM ΔMAP; HEM: −58.4±7.2 mm Hg vs. saline: −3.3±1.9 mm Hg, P<0.001, using the Independent sample t-test, n=6). Mean±SEM ΔHR increased but it was not significant (HEM: −10.6±9.3 beats/min vs. saline: −7.1±5.4 beats/min).

Twenty minutes after termination of blood withdrawal, cardiovascular parameters were stabilized but was lower than those in the control group (Mean±SEM ΔSBP; HEM: −31.6±3.7 mm Hg vs. saline: −4.6±2.3 mm Hg, P<0.01) and (Mean±SEM ΔMAP; HEM: −28.9±3.3 mm Hg vs. saline: −3.3±1.9 mm Hg, P<0.01). Mean±SEM ΔHR significantly increased compared to that in the control group (HEM: 86.4±17.4 mm Hg vs. saline: −7.1±5.4; P<0.001; using the Independent sample t-test) (Figures 3 and 4).

### 3.3. Cardiovascular responses of PPT to microinjection of saline and cobalt(II) chloride before Hemorrhage

In these experiments, first, saline and CoCl_2_ in separate groups were microinjected into the PPT and after 5 min the blood withdrawal was performed (Figure 3). Time-course changes of SBP, MAP, and HR after Hemorrhage are shown in Figure 4. As it is shown, in HEM+saline group, all parameters were not significant compared to those of the HEM group but were significant compared to those of the control group (using repeated measures ANOVA, P<0.001) (Figure 4). In CoCl_2_+HEM group, changes of MAP and SBP in several time points were significantly more than those in the HEM group and changes of HR was significantly lower than that in the HEM group (using repeated measures ANOVA, P<0.001) (Figure 4).

The peak ΔSBP, ΔMAP, and ΔHR in 0 and 20 min after termination of blood withdrawal in saline+HEM and CoCl_2_+HEM groups were also evaluated. As it is shown in Figure 4 a and 4 b, immediately after termination of blood withdrawal, Mean±SEM ΔSBP and ΔMAP were not significant in saline+HEM group compared to those in the HEM group, but was significant in comparison with those in the control group (ΔSBP in HEM+saline: −60.7±5.8 mm Hg vs. saline: −4.6±2.3 mm Hg; P<0.001 and ΔMAP in HEM+saline: −51.8±6.4 mm Hg vs. saline: −3.3±1.9 mm Hg; P<0.001). Peak ΔHR in this time did not change compared to both HEM and control groups (HEM+saline: 20.3±11.5 beats/min vs. saline: −0.1±5.4 beats/min) (Figure 4 c).

In CoCl_2_+HEM group, Mean±SEM ΔSBP and ΔMAP were significantly decreased immediately after blood withdrawal compared to HEM group (ΔSBP in CoCl_2_+HEM: −43.4±6.4 mm Hg vs. HEM: −64.7±7.9 mm Hg; P<0.001 (Figure 4 a) and ΔMAP in CoCl_2_+HEM: −31.4±5.7 mm Hg vs. HEM: −58.4±7.2 mm Hg, P<0.001 (Figure 4 b). In this time, ΔHR decreased but it was not significant compared to that in HEM group (HEM+CoCl_2_: 20.3±8.9 beats/min vs. saline: −10.6±9.3 beats/min) (Figure 4 c).

Investigating cardiovascular parameters 20 min after termination of blood withdrawal indicates that in saline+HEM group, ΔSBP and ΔMAP were not significant compared to those in the HEM group but was lower than those in the control group (Mean±SEM ΔSBP in HEM+saline: −28.4±4.6 mm Hg vs. saline −4.6±2.3 mm Hg, P<0.01 (Figure 4 a) and Mean±SEM ΔMAP: −25.7±4.1 mm Hg vs. control: −3.3±1.9 mm Hg, P<0.01 (Figure 4 b). Mean±SEM ΔHR was significant in comparison with that in the control group (HEM+saline: 77.4±15.8 beats/min vs. saline: −7.1±5.4 beats/min; P<0.001) (Figure 4 c).

In HEM+CoCl_2_ group, 20 min after termination of blood withdrawal, Mean±SEM ΔSBP and ΔMAP significantly increased compared to those in the HEM group (ΔSBP in HEM+CoCl_2_; −17.1±2.2 mm Hg vs. HEM: −36.6±3.7 mm Hg; P<0.05 (Figure 4 a) and (ΔMAP in HEM+CoCl_2_: −11.2±4.4 mm Hg vs. HEM: −33.9±3.3 mm Hg; P<0.05 (Figure 4 b). ΔHR also significantly decreased compared to HEM group (HEM+CoCl_2_: 18.6±10.5 beats/min vs. HEM: 86.4±17.4 beats/min, P<0.01) (Figure 4 c).

## 4. Discussion

The present study indicates that inactivation of PPT with CoCl_2_ does not affect normal cardiovascular parameters. However, inactivation of this nucleus during Hemorrhage significantly reduced the hypotension and tachycardia induced by Hemorrhage. CoCl_2_ is a non-selective blocker of the pre-synaptic Ca^2+^ influx that by inhibiting the release of neurotransmitter could inactivate the brain areas and allow researchers to recognize the physiologic function of a nucleus (Plowey & Waldrop, 2004).

Our results indicate that inactivation of PPT with CoCl_2_ does not change cardiovascular parameters. This result indicates that vasomotor neurons of the PPT are not active in normal condition. The CNS plays an important role in the regulation of the cardiovascular system during the normal condition. The cardiovascular effect of CNS is mostly regulated by the sympathetic nervous system (Dampney et al., 2003).

The sympathetic nervous system is regulated by several supraspinal areas such as RVLM, hypothalamus nuclei, and NTS (Dampney, 1994). RVLM is an important sympathoexcitatory area that via a direct projection to sympathetic preganglionic neurons in the spinal cord, contributes to the regulation of the cardiovascular system (Dampney, 1994). RVLM area has intrinsic activity and also receives numerous inputs from other areas to regulate the cardiovascular system during the resting condition (Guyenet, 2006).

Because inactivation of PPT does not have significant effects on resting cardiovascular parameters, it is conceivable that neurons related to PPT cardiovascular effect are not important in the resting regulation of the cardiovascular system. Although PPT does not involve in the resting cardiovascular function, it is active in conditions such as exercise and locomotion (Takakusaki, 2008). Therefore, it is possible that PPT neurons related to cardiovascular regulation in resting condition are inhibited by other neurons of the PPT. Consistent with this theory, our results indicate that the nitrergic (Shafei et al., 2017) and cholinergic neurons of the PPT (Nikyar, Hosseini, Niazmand, & Shafei, 2018) have an inhibitory effect on the cardiovascular system. In addition, PPT has a feedforward action that regulates cardiovascular responses to locomotion and exercise (Padley et al., 2007).

It confirms that neurons of this nucleus are mostly active during physiologic functions such as exercise. Based on this theory, in normal condition, the activity of PPT neuron(s) related to control of the cardiovascular system is quiescent or has very low activity. Therefore the blockade of the PPT by CoCl_2_ could not change basic cardiovascular parameters.

In the second experiment, to evaluate the involvement of the PPT nucleus in hypotension induced by Hemorrhage, CoCl_2_ was microinjected into the PPT nucleus 5 minutes before the induction of Hemorrhage. The result showed that inactivation of the nucleus could inhibit hypotension and tachycardia induced by Hemorrhage. Based on this effect, we suggest that neurons of the PPT are involved in the regulation of the cardiovascular response during Hemorrhage.

Hemorrhage is a threatening condition that initiates a complex of neural and humoral responses. Response to Hemorrhage has two compensatory and non-compensatory phases. In the compensatory phase, the sympathetic nervous system is activated and through peripheral vasoconstriction and tachycardia keeps the blood pressure constant despite the decreased blood volume. If the blood loss continues, non-compensatory phase occurs, where the activity of the sympathetic nervous system decreases and the blood pressure abruptly falls. In this experiment, our goal was to keep the animal in a compensatory phase (Schadt & Ludbrook, 1991).

Our results also showed that after the beginning of the blood withdrawal, the blood pressure and Heart Rate decreased, but afterward the blood pressure drop gradually reversed and was stabilized at 30–40 mm Hg lower than normal condition but the Heart Rate increased and was significantly higher than normal. Inactivation of PPT with CoCl_2_ attenuates hypotension and tachycardia response induced by Hemorrhage that indicates the involvement of PPT in the modulation of cardiovascular parameters during Hemorrhage. The exact underlying mechanism(s) of this effect is unknown but several hypotheses could be suggested.

PPT has projections to several areas which are involved in cardiovascular regulation such as RVLM, NTS, and vlPAG (Steininger et al., 1992). Projection of PPT into RVLM has been reported in the previous study. Our previous study also shows that the nitrergic system and the cholinergic system of the PPT inhibit cardiovascular system (Shafei et al., 2017). Therefore it is conceivable that PPT-RVLM is involved in the modulation of cardiovascular during Hemorrhage. Anatomical relationship between PPT and NTS has been previously reported (Topchiy et al., 2010).

NTS plays a pivotal role in the regulation of baroreflex and chemoreflex activity. Because Hemorrhage elicits both baroreflex and chemoreflex via NTS, we suggest that the relation of PPT with NTS may be involved in the modulation of baroreflex and chemoreflex during Hemorrhage. In addition, there is evidence that PPT has a relation with vlPAG nucleus (Steininger et al., 1992) that is involved in the integration of cardiovascular response to Hemorrhage (Cavun & Millington, 2001).

The presence of several neurotransmitters such as acetylcholine, gamma aminobutyric acid (Martinez-Gonzalez et al., 2011), and serotonin (Strecker et al., 1999) also has been shown in this nucleus. All of these neurotransmitters contribute in cardiovascular responses during Hemorrhage (Altinbas, Yilmaz, Savci, Jochem, & Yalcin, 2015; Kung, Glasgow, Ruszaj, Gray, & Scrogin, 2010; Tanaka, Miyakubo, Hayashi, & Nomura, 2001).

It is possible that these neurotransmitters contribute to the response of the PPT toward Hemorrhage. In this study, HR increased during the Hemorrhage and inactivation of the PPT with CoCl_2_ attenuates tachycardia induced by Hemorrhage that supports the involvement of this nucleus in tachycardia response during Hemorrhage. The effect of the PPT on HR may be mediated via the relationship of this nucleus with NTS or direct effect on the vagal system. However, in the present time, the underlying mechanism(s) of the PPT effect on Hemorrhage is unknown and calls for future studies to clarify this effect of PPT nucleus.

In brief, our results indicate that inactivation of PPT by CoCl_2_ does not have any effects on normal state cardiovascular parameters but during Hemorrhage, the inactivation of this nucleus facilitates the recovery of blood pressure and attenuates tachycardia induced by Hemorrhage. However future studies are needed to evaluate these findings.

## References

[B1] Ahlgren J. Porter K. Hayward L. F. (2007). Hemodynamic responses and c-Fos changes associated with hypotensive Hemorrhage: Standardizing a protocol for severe Hemorrhage in conscious rats. American Journal of Physiology-Regulatory, Integrative and Comparative Physiology, 292(5), R1862–71. [DOI:10.1152/ajpregu.00325.2006] [PMID ] 17218446

[B2] Altinbas B. Yilmaz M. S. Savci V. Jochem J. Yalcin M. (2015). Centrally injected histamine increases posterior hypothalamic acetylcholine release in Hemorrhage-hypotensive rats. Autonomic Neuroscience, 187, 63–9. [DOI:10.1016/j.aut-neu.2014.11.004] [PMID ] 25468497

[B3] Benarroch E. E. (2013). Pedunculopontine nucleus functional organization and clinical implications. Neurology, 80(12), 1148–55. [DOI:10.1212/WNL.0b013e3182886a76] [PMID ] 23509047

[B4] Bevan M. Bolam J. (1995). Cholinergic, GABAergic, and glutamate-enriched inputs from the mesopontine tegmentum to the subthalamic nucleus in the rat. The Journal of Neuroscience, 15(11), 7105–20. [DOI:10.1523/JNEUROSCI.15-11-07105.1995] [PMID ] 7472465 PMC6578076

[B5] Cavun S. Millington W. R. (2001). Evidence that hemorrhagic hypotension is mediated by the ventrolateral periaqueductal gray region. American Journal of Physiology-Regulatory, Integrative and Comparative Physiology, 281(3), R747–52. [DOI:10.1152/ajpregu.2001.281.3.R747] [PMID ] 11506988

[B6] Dampney R. (1994). Functional organization of central pathways regulating the cardiovascular system. Physiological Reviews, 74(2), 323–64. [DOI:10.1152/physrev.1994.74.2.323] [PMID ] 8171117

[B7] Dampney R. Horiuchi J. Tagawa T. Fontes M. Potts P. Polson J. (2003). Medullary and supramedullary mechanisms regulating sympathetic vasomotor tone. Acta Physiologica, 177(3), 209–18. [DOI:10.1046/j.1365-201X.2003.01070.x] [PMID ] 12608991

[B8] Evans R. G. Ventura S. Dampney R. A. L. Ludbrook J. (2001). John Ludbrook APPS symposium neural mechanisms in the cardiovascular responses to acute central hypovolaemia. Clinical and Experimental Pharmacology and Physiology, 28(5–6), 479–87. [DOI:10.1046/j.1440-1681.2001.3473.x] 11428384

[B9] Granjeiro E. M. Gomes F. V. Alves F. H. Crestani C. C. Corrêa F. M. Resstel L. B. (2012). Bed nucleus of the stria terminalis and the cardiovascular responses to chemoreflex activation. Autonomic Neuroscience: Basic and Clinical, 167(1), 21–6. [DOI:10.1016/j.autneu.2011.11.004] [PMID ] 22197162

[B10] Guyenet P. G. (2006). The sympathetic control of blood pressure. Nature Reviews Neuroscience, 7(5), 335–46. [DOI:10.1038/nrn1902] [PMID ] 16760914

[B11] Kung L. H. Glasgow J. Ruszaj A. Gray T. Scrogin K. E. (2010). Serotonin neurons of the caudal raphe nuclei contribute to sympathetic recovery following hypotensive Hemorrhage. American Journal of Physiology-Regulatory, Integrative and Comparative Physiology, 298(4), R939–53. [DOI:10.1152/ajpregu.00738.2009] [PMID ] [PMCID ] PMC2853390 20130223

[B12] Martinez-Gonzalez C. Bolam J. P. Mena-Segovia J. (2011). Topographical organization of the pedunculopontine nucleus. Frontiers in Neuroanatomy, 5, 5–22. [DOI:10.3389/fnana.2011.00022] [PMID ] [PMCID ] 21503154 PMC3074429

[B13] Nasimi A. Shafei M. N. Alaei H. (2012). Glutamate injection into the cuneiform nucleus in rat, produces correlated single unit activities in the Kolliker-Fuse nucleus and cardiovascular responses. Neuroscience, 223, 439–46. [DOI:10.1016/j.neuroscience.2012.07.041] [PMID ] 22858597

[B14] Nikyar T. Hosseini M. Niazmand S. Shafei M. N. (2018). Evaluation of nicotinic receptor of Pedunculopontine Tegmental nucleus in central cardiovascular regulation in anesthetized rat. Iranian Journal of Basic Medical Sciences, 21(4), 376–81. [DOI:10.22038/IJBMS.2018.25616.6319] [PMID ] [PMCID ] 29796220 PMC5960753

[B15] Padley J. R. Kumar N. N. Li Q. Nguyen T. B. Pilowsky P. M. Goodchild A. K. (2007). Central command regulation of circulatory function mediated by descending pontine cholinergic inputs to sympathoexcitatory rostral ventrolateral medulla neurons. Circulation Research, 100(2), 284–91. [DOI:10.1161/01.RES.0000257370.63694.73] [PMID ] 17204655

[B16] Pahapill P. A. Lozano A. M. (2000). The pedunculopontine nucleus and Parkinson’s disease. Brain, 123(9), 1767–83. [DOI:10.1093/brain/123.9.1767] [PMID ] 10960043

[B17] Paxinos G. Watson C. H. (2007). The rat brain in stereotaxic coordinates. New York: Academic Press. 10.1016/0165-0270(80)90021-7 6110810

[B18] Plowey E. D. Waldrop T. G. (2004). Cobalt injections into the pedunculopontine nuclei attenuate the reflex diaphragmatic responses to muscle contraction in rats. Journal of Applied Physiology, 96(1), 301–7. [DOI:10.1152/japplphysiol.00652.2003] [PMID ] 12972440

[B19] Schadt J. C. Ludbrook J. (1991). Hemodynamic and neurohumoral responses to acute hypovolemia in conscious mammals. American Journal of Physiology-Heart and Circulatory Physiology, 260(2), H305–18. [DOI:10.1152/ajpheart.1991.260.2.H305] [PMID ] 1671735

[B20] Shafei M. N. Nasimi A. (2011). Effect of glutamate stimulation of the cuneiform nucleus on cardiovascular regulation in anesthetized rats: Role of the pontine Kolliker-Fuse nucleus. Brain Research, 1385, 135–43. [DOI:10.1016/j.brain-res.2011.02.046] [PMID ] 21349254

[B21] Shafei M. N. Nikyar T. Hosseini M. Niazmand S. Paseban M. (2017). Cardiovascular effects of nitrergic system of the Pedunculopontine Tegmental nucleus in anesthetized rats. Iranian Journal of Basic Medical Sciences, 20(7), 776–82. [DOI:10.22038/IJBMS.2017.9009] [PMCID ] [PMID ] 28852442 PMC5569585

[B22] Sleekier T. Inglis W. Winn P. Sahgal A. (1994). The Pedunculopontine Tegmental nucleus: A role in cognitive processes. Brain Research Reviews, 19(3), 298–318. [DOI:10.1016/0165-0173(94)90016-7] 7820134

[B23] Steininger T. L. Rye D. B. Wainer B. H. (1992). Afferent projections to the cholinergic Pedunculopontine Tegmental nucleus and adjacent midbrain extrapyramidal area in the albino rat: I. Retrograde tracing studies. Journal of Comparative Neurology, 321(4), 515–43. [DOI:10.1002/cne.903210403] [PMID ] 1380518

[B24] Strecker R. E. Thakkar M. M. Porkka-Heiskanen T. Dauphin L. J. Bjorkum A. McCarley R. W. (1999). Behavioral state-related changes of extracellular serotonin concentration in the Pedunculopontine Tegmental nucleus: A microdialysis study in freely moving animals. Sleep Research Online, 2(2), 21–27. [PMID ] 11421239

[B25] Takakusaki K. (2008). Forebrain control of locomotor behaviors. Brain Research Reviews, 57(1), 192–8. [DOI:10.1016/j.brainres-rev.2007.06.024] [PMID ] 17764749

[B26] Tanaka J. Miyakubo H. Hayashi Y. Nomura M. (2001). Hemorrhage activates catecholaminergic neurons sensitive to GABA in the nucleus of the solitary tract with ascending projections to the subfornical organ in rats. Autonomic Neuroscience, 91(1), 100–4. [DOI:10.1016/S1566-0702(01)00294-6] 11515796

[B27] Topchiy I. Waxman J. Radulovacki M. Carley D. W. (2010). Functional topography of respiratory, cardiovascular and pontine-wave responses to glutamate microstimulation of the pedunculopontine tegmentum of the rat. Respiratory Physiology & Neurobiology, 173(1), 64–70. [DOI:10.1016/j.resp.2010.06.006] [PMID ] [PMCID ] 20601208 PMC2979313

[B28] Yasui Y. Cechetto D. F. Saper C. B. (1990). Evidence for a cholinergic projection from the Pedunculopontine Tegmental nucleus to the rostral ventrolateral medulla in the rat. Brain Research, 517(1), 19–24. [DOI:10.1016/0006-8993(90)91002-X] 2375988

